# Social stress shortens lifespan in mice

**DOI:** 10.1111/acel.12778

**Published:** 2018-05-28

**Authors:** Maria Razzoli, Kewir Nyuyki‐Dufe, Allison Gurney, Connor Erickson, Jacob McCallum, Nicholas Spielman, Marta Marzullo, Jessica Patricelli, Morito Kurata, Emily A. Pope, Chadi Touma, Rupert Palme, David A. Largaespada, David B. Allison, Alessandro Bartolomucci

**Affiliations:** ^1^ Department of Integrative Biology and Physiology University of Minnesota Minneapolis Minnesota; ^2^ Department of Pediatric and Masonic Cancer Center University of Minnesota Minneapolis Minnesota; ^3^ Department of Behavioural Biology University of Osnabrück Osnabrück Germany; ^4^ Department of Biomedical Sciences University of Veterinary Medicine Vienna Austria; ^5^ School of Public Health Indiana University – Bloomington Bloomington Indiana

**Keywords:** aggression, atherosclerosis, healthspan, lifespan, senescence, stress

## Abstract

Stress and low socioeconomic status in humans confer increased vulnerability to morbidity and mortality. However, this association is not mechanistically understood nor has its causation been explored in animal models thus far. Recently, cellular senescence has been suggested as a potential mechanism linking lifelong stress to age‐related diseases and shorter life expectancy in humans. Here, we established a causal role for lifelong social stress on shortening lifespan and increasing the risk of cardiovascular disease in mice. Specifically, we developed a lifelong chronic psychosocial stress model in which male mouse aggressive behavior is used to study the impact of negative social confrontations on healthspan and lifespan. C57BL/6J mice identified through unbiased cluster analysis for receiving high while exhibiting low aggression, or identified as subordinate based on an ethologic criterion, had lower median and maximal lifespan, and developed earlier onset of several organ pathologies in the presence of a cellular senescence signature. Critically, subordinate mice developed spontaneous early‐stage atherosclerotic lesions of the aortic sinuses characterized by significant immune cells infiltration and sporadic rupture and calcification, none of which was found in dominant subjects. In conclusion, we present here the first rodent model to study and mechanistically dissect the impact of chronic stress on lifespan and disease of aging. These data highlight a conserved role for social stress and low social status on shortening lifespan and increasing the risk of cardiovascular disease in mammals and identify a potential mechanistic link for this complex phenomenon.

## 1 INTRODUCTION

Classical clinical and epidemiological studies unequivocally demonstrate that stress, allostatic load, low socioeconomic status (SES), and low social rank confer increased vulnerability to morbidity significantly shortening lifespan in humans (Chetty et al., 2016; Marmot et al., 1991; Seeman, Singer, Rowe, Horwitz & McEwen, 1997; Stringhini et al., 2012). Notably, a recent multicohort study of 1.7 million adults followed up for mortality (Stringhini et al., 2017) has recognized the effect of SES among other recognized WHO risk factors (i.e., smoking, obesity, diabetes, hypertension, alcohol intake) assigning it a notable 1.26 hazard ratio. Similarly, an association has been demonstrated between psychological stress, senescence as measured by telomere shortening, and increased mortality (Epel & Lithgow, 2014; Epel et al., 2004). Altogether, these evidences support the inclusion of failed “adaptation to stress,” as one of the so‐called *Seven Pillars of Aging* (Kennedy et al., 2014). Similar to human condition, low social rank and sustained lifetime stress significantly worsen healthspan and increase mortality rate in nonhuman primates (Maestripieri & Hoffman, 2011; Sapolsky, 2005; Tung, Archie, Altmann & Alberts, 2016). Classical studies in rodents housed in seminatural conditions (Calhoun, 1962; Henry & Stephens, 1977) also showed that high population density corresponded to a high number of animals showing signs of stress pathology, including cardiovascular disease, leading to high mortality. Since then, rodent models manipulating social stress and social rank have been developed in young, adult, and mature individuals, causally linking chronic stress to disparate pathologies (Bale & Epperson, 2015; Krishnan et al., 2007; Lassance‐Soares et al., 2014; Pryce & Fuchs, 2017; Scharf, Sterlemann, Liebl, Müller & Schmidt, 2013).

Overall, data in human and animal models call attention to the urgent need to understand how different stressors accelerate and/or offset aging and associated chronic diseases (Childs et al., 2017; Gil & Withers, 2016; López‐Otín, Blasco, Partridge, Serrano & Kroemer, 2013). Nevertheless, in spite of available evidences on the mechanisms regulating aging and stress responses (Koolhaas et al., 2011; McEwen, 2007; Prather et al., 2015) and the profound effect of stress on pathophysiology (Bartolomucci, 2007; McEwen, 2007), this association is not mechanistically understood nor has its causation been explored in animal models thus far.

Here, we developed a model of lifelong chronic psychosocial stress (LCPS) in male mice, which allows identification of a causal role for subordination stress on shortening lifespan and increasing cardiovascular disease risk in mice. Our results demonstrate that LCPS mice characterized by receiving high while exhibiting low aggression or identified as subordinate (equivalent to low social rank, a model of low SES) showed lower survival probability and increased markers of cellular senescence compared to dominant mice. Remarkably, subordinate, but not dominant mice, developed spontaneous early‐stage atherosclerotic lesions. Our model is based on the established vulnerability of male mice to chronic psychosocial stress (Bartolomucci, 2007). Female laboratory mice do not manifest a territorial aggression comparable to males and are generally more resilient than males to most common models of social stress (Palanza, 2001). Thus, further studies are required to extend the validity of our results to female mice.

Our findings demonstrate a conserved role for the negative impact of social stress on survival implicating mechanisms of increased risk of cardiovascular disease, while providing a functional explanation to epidemiological and experimental human data as well as a biological platform to investigate the impact of psychosocial stress on aging‐related diseases and survival.

## 2 RESULTS

### 2.1 Low survival probability in mice receiving high aggression while manifesting a passive coping style

**Figure 1:**
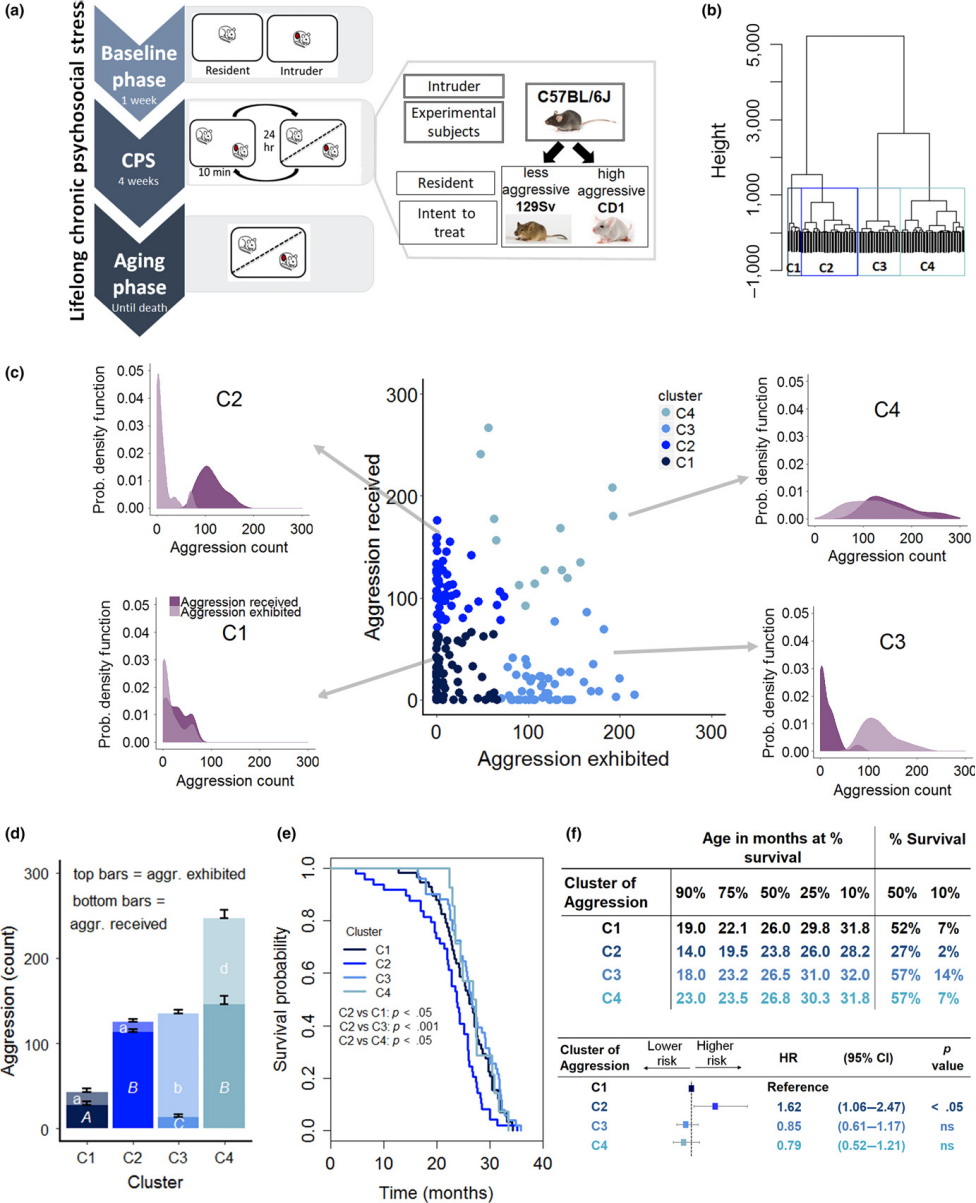
Effect of contextual aggression on survival. (a) Diagram of the lifelong chronic psychosocial stress (LCPS) model. (b) Dendrogram of the experimental population based on aggression exhibited and received by experimental subjects in the course of the 4‐week CPS phase and the statistically identified clusters (C1, *N* = 58, C2, *N* = 49, C3, *N* = 51, C4, *N* = 14). (c) Scatterplot of the amount of aggression exhibited and received by experimental subjects during the 4‐week CPS highlighting the four statistically identified clusters and associated individual probability density plots that exemplify the probability of receiving and exhibiting a given amount of aggression by individuals within each cluster. (d) Average amount of aggression exhibited [*F*(3,142) = 184.76, *p* < .0001, different letters represent statistically significant binary differences at post hoc level] and received [*F*(3,142) = 188.06, *p* < .0001] by the four clusters. (e) Survival probability as affected by aggression cluster (log‐rank test, χ^2^ = 13.6 on 3 degrees of freedom, *p* < .01; Bonferroni corrected *p* value for binary comparisons = 0.0083). (f) Age at which 90%, etc. of the population survived, the percent survival at median and maximum (10%) survival as well as Cox regression model examining the contribution of cluster of aggression or achieved status on survival. (likelihood ratio test = 11.36 on 3 degrees of freedom, *p* = .009915: C2 vs. C1 *p* = .024, C3 vs. C1, ns, C4 vs. C1, ns) (HR, hazard ratio; CI, confidence interval)

Our working hypothesis was that the degree of aggression exhibited/received and the achieved social status would impact healthspan and lifespan in mice analogous to the effect of SES on disease susceptibility and life expectancy in humans. To test this hypothesis, we developed a lifelong chronic psychosocial stress (LCPS) model. Operationally, for the purpose of this study, a 4‐week long *CPS phase*, characterized by daily aggressive interaction and sensory (sight, sound, and smell) cohousing, was followed by an *aging phase*, characterized by lifelong sensory housing in the absence of overt aggressive interactions (Figure 1a). C57BL/6J male mice were randomly paired to resident males of either the CD1 or the Sv129Ev strain to attain exposure to a gradient of territorial aggression, reportedly higher in CD1 (Bartolomucci et al., 2010; Dadomo et al., 2011; Zou, Storm & Xia, 2013). High‐fat diet (HFD) was previously reported to exacerbate stress‐induced metabolic syndrome‐like (Sanghez et al., 2013) and to shorten lifespan (Baur et al., 2006), prompting us to test the hypothesis that there could be an interaction between stress and hypercaloric diet on survival. Thus, half the mice were randomized to HFD feeding during the last 3 weeks of the *CPS phase* according to our established protocol (Razzoli et al., 2016; Sanghez et al., 2013, 2016). HFD was limited to 3 weeks as chronic HFD per se has a marked effect on lifespan (Baur et al., 2006). The *aging phase* started subsequently by maintaining the two pair members cohoused in sensory contact until spontaneous death, thus mimicking a lifelong stress threat.

Mice were stratified according to their aggressive behavior by applying an unbiased cluster analysis to aggression exhibited/received during the CPS phase that identified four clusters (C) (Figure 1b–d). *Cluster 1* (C1, *N* = 58) was composed of individuals that received and exhibited low aggression (Figure 1d) (similar to standard group housing); *C2* (*N* = 49) was composed of mice that received high levels of aggression while exhibiting little aggression (similar to animals of low in social rank); *C3* (*N* = 51) subjects received low aggression while themselves expressing high amount of agonistic behaviors (similar to animals of high social rank); finally, *C4* (*N* = 14) was composed of individuals receiving and exhibiting high levels of aggression (typical of unstable social hierarchies) (Brain & Parmigiani, 1990). As evident in Figure 1c, the frequency of subjects within each cluster exhibiting or experiencing a given level of aggression was largely overlapping for *C1* or *C4*, while they were opposite in *C2* and *C3*.

Remarkably, this contextual aggression‐dependent clustering corresponded to a very striking difference in survival outcome. Subjects receiving high aggression and having an otherwise passive coping style in terms of aggression exhibited (*C2*) had the worst survival outcome and a significant increase in the hazard ratio compared to all the other clusters (Figure 1e–f).

Notably, individuals experiencing either low overall aggression similar to standard group housing (*C1*), or high interindividual aggression (*C4*), or establishing a clear pattern of social dominance (*C3*), had overlapping survival trajectories and hazard ratios (Figure 1e–f) that also matched the expected survival probability for this strain (Mitchell et al., 2014; Turturro et al., 1999). Conversely, our a priori experimental design‐included factors such as pairing with a high/low aggressive strain or high/standard fat diet that had no effect on mouse survival (Figure S1a–b). Our results reveal that low survival probability is not caused by the absolute amount of aggression received unless this is experienced by an animal that does not reciprocate but rather manifests a passive coping style.

### 2.2 Subordination stress shortens lifespan in mice

**Figure 2:**
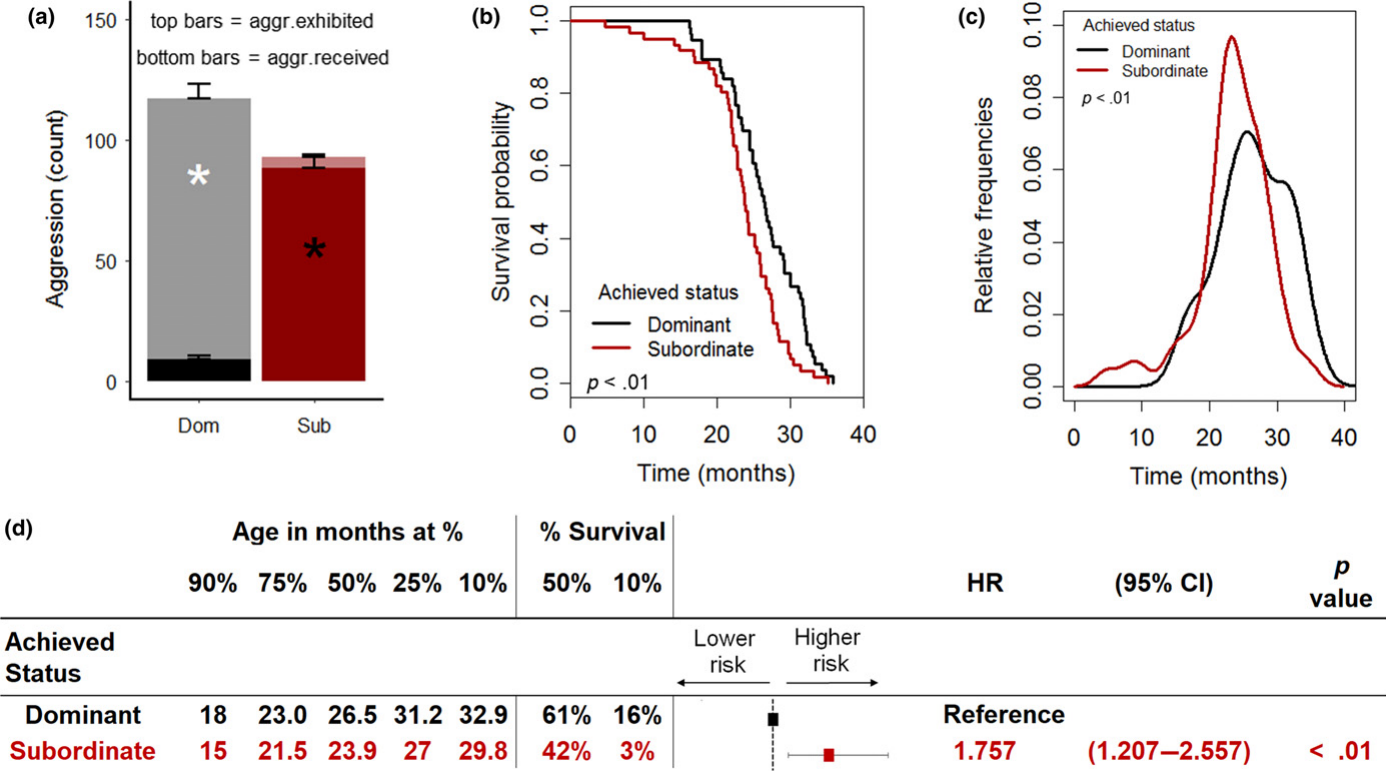
Achieved subordinate status shortens lifespan in mice. (a) Average amount of aggression exhibited [*F*(1,115) = 309.35, *p* < .0001)] and received [*F*(1,115) = 176.1, *p* < .0001] by individual according to the achieved status (subordinate = 61, dominant = 56). (b, c) Survival probability and distribution of mortality frequencies as affected by the achieved status (log‐rank test, χ^2^ = 8.9 on 1 degrees of freedom, *p* < .01). (d) Age at which 90%, etc. of the population survived, the percent survival at median and maximum (10%) survival as well as Cox regression model examining the contribution of achieved status on survival (HR, hazard ratio; CI, confidence interval). *p < .05

One critical finding of our study is that *Cluster 2* and *Cluster 3* had opposite distributions of aggression received/exhibited representative of animals belonging to a low and high social rank, respectively, according to the classical ethological definition of social hierarchy (Bartolomucci, 2007; Sapolsky, 2005). Thus, we reanalyzed our dataset based on the more restrictive standard definition of achieved dominant and subordinate social status (see methods; Figure 2a) previously shown to explain individual vulnerability to cardiovascular (Costoli et al., 2004), metabolic (Sanghez et al., 2013), and psychiatric (Bartolomucci et al., 2010) disorders.

Subordinate mice had a significantly shorter lifespan compared to dominant animals (Figures 2b and S1c), with the distribution of frequency of age at death that peaked earlier than for dominant animals (Figure 2c). The curve describing the survival probability of subordinate mice corresponded to a 12.4% decrease in the median lifespan and to a significant decline in the maximal lifespan compared to dominant mice (Figure 2b,d). Subordinate status corresponded to a significant hazard ratio of 1.75 compared to being dominant (Figure 2d). The negative impact of the achieved subordinate status on survival did not interact with the diet (Figure S1d).

Overall, the difference in survival between subordinate and dominant groups largely reproduced the difference between the *C2* and the *C3* groups (Figure 1e). Importantly, the lifespan of the dominant group was comparable to the *C1*,* C3*, and *C4* groups (Figure 1e,f) and to the expected survival rate for the C57BL/6J strain thus suggesting that dominant could be considered a reference group for aging‐associated conditions in our study. Overall, these results demonstrate that the low survival probability is an intrinsic characteristic of animals achieving a subordinate status.

### 2.3 Subordination stress impairs healthspan in mice

**Figure 3:**
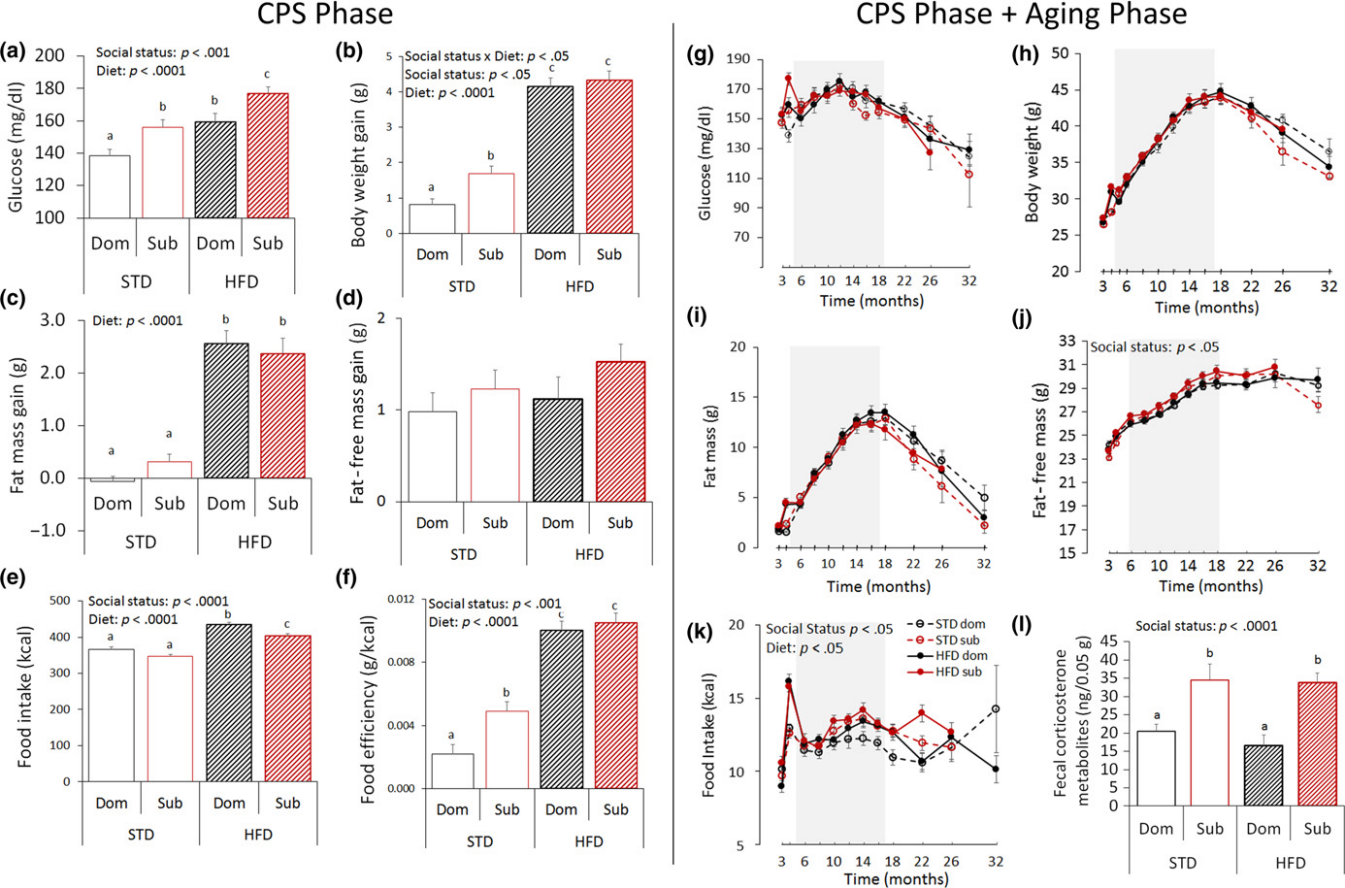
Achieved status impacts healthspan in mice. (a–f) Healthspan parameters during the 4 weeks of chronic psychosocial stress (CPS) exposure: (a) 4‐hr fasting glucose [diet: *F*(1,112) = 20.82, *p* < .0001; achieved status: *F*(1,112) = 14.59, *p* < .001]. (b) body weight gain [diet: *F*(1,113) = 186.71, *p* < .0001; achieved status: *F*(1,113) = 4.21, *p* < .05], (c) fat mass gain [diet: *F*(1,113) = 132.56, *p* < .0001]; (d) fat‐free mass gain; (e) food intake [diet: *F*(1,113) = 111.16, *p* < .0001; achieved status: *F*(1,113) = 12.12, *p* < .0001]; (f) food efficiency [diet: *F*(1,113) = 134.26, *p* < .0001; achieved status: *F*(1,113) = 7.90, *p* < .01]; (g–l) Healthspan parameters during the entire duration of the study: (g) 4‐hr fasting glucose after; (h) body weight; (i) fat mass; (j) fat‐free mass [achieved status: *F*(1,108) = 5.73, *p* < .05]; (k) food intake [diet: *F*(1,107) = 4.41, *p* < .05; achieved status: *F*(1,107) = 6.62, *p* < .05]; (STD**,** standard diet; HFD**,** high‐fat diet). While data are presented for the entire study duration (g–k), they were statistically analyzed only until 12 months after stress (shaded areas), a point upon which the number of missing samples started exceeding 5% thus affecting the analytical power [78]; missing data from animals not surviving to 12 months after stress (3.76%) were not treated nor included in this analysis. Different letters represent statistical differences between groups. (l) Fecal corticosterone metabolites [*F*(1,50) = 19.8, *p* < .0001]. *N* = 8/group. *p < .05, #*p* < .06. Data represent group mean** **±** **SEM

To gain insights on the life trajectories produced by the LCPS model, healthspan assessments were conducted regularly throughout the duration of the study (Figure 3). During the CPS phase, subordinate mice developed hyperglycemia when fed both a STD or a HFD (Figure 3a), a finding which is consistent with our previous work (Sanghez et al., 2013) and that could have set the stage for a later metabolic dysregulation and impact later survival outcomes (Fink, Kolterman, Griffin & Olefsky, 1983; Houtkooper et al., 2011). Subordinate mice were also characterized by increased body weight when fed a STD (Figure 3b–d) despite eating comparably to dominant mice (Figure 3e) that resulted in a greater food efficiency (Figure 3f). The vulnerability to HFD‐induced obesity was similar among the two groups probably due to the large hyperphagia shown by the dominant mice (Figure 3b,c). This finding in the C57BL/6J strain is at variance with previous data obtained in CD1 mice (Sanghez et al., 2013; Bartolomucci et al., 2009) thus suggesting a strain difference in metabolism (Fengler et al., 2016; Zou et al., 2013).

During the aging phase, the effect of HFD on metabolism vanished rapidly after its discontinuation (Figure 3g–k). Nevertheless, subordinate mice developed a significant lifelong hyperphagia (Figure 3k) and increased fat‐free mass (Figure 3j) compared to dominant mice. Subordinate mice also showed elevated levels of fecal corticosterone metabolites (measured after 2 months of the aging phase) compared to dominant mice (Figure 3l), suggesting sustained activation of the HPA axis as a consequence of the achieved status. Overall, these data demonstrate a significant and long‐lasting effect of the achieved subordinate status on mice healthspan. As the metabolic effects of HFD vanished after its discontinuation, diet as an experimental factor was not included in subsequent pathological and biomarker analyses conducted on 17‐months old mice (see below).

### 2.4 Subordination stress induced an earlier onset of aging‐associated diseases and increased cellular markers of senescence

**Figure 4:**
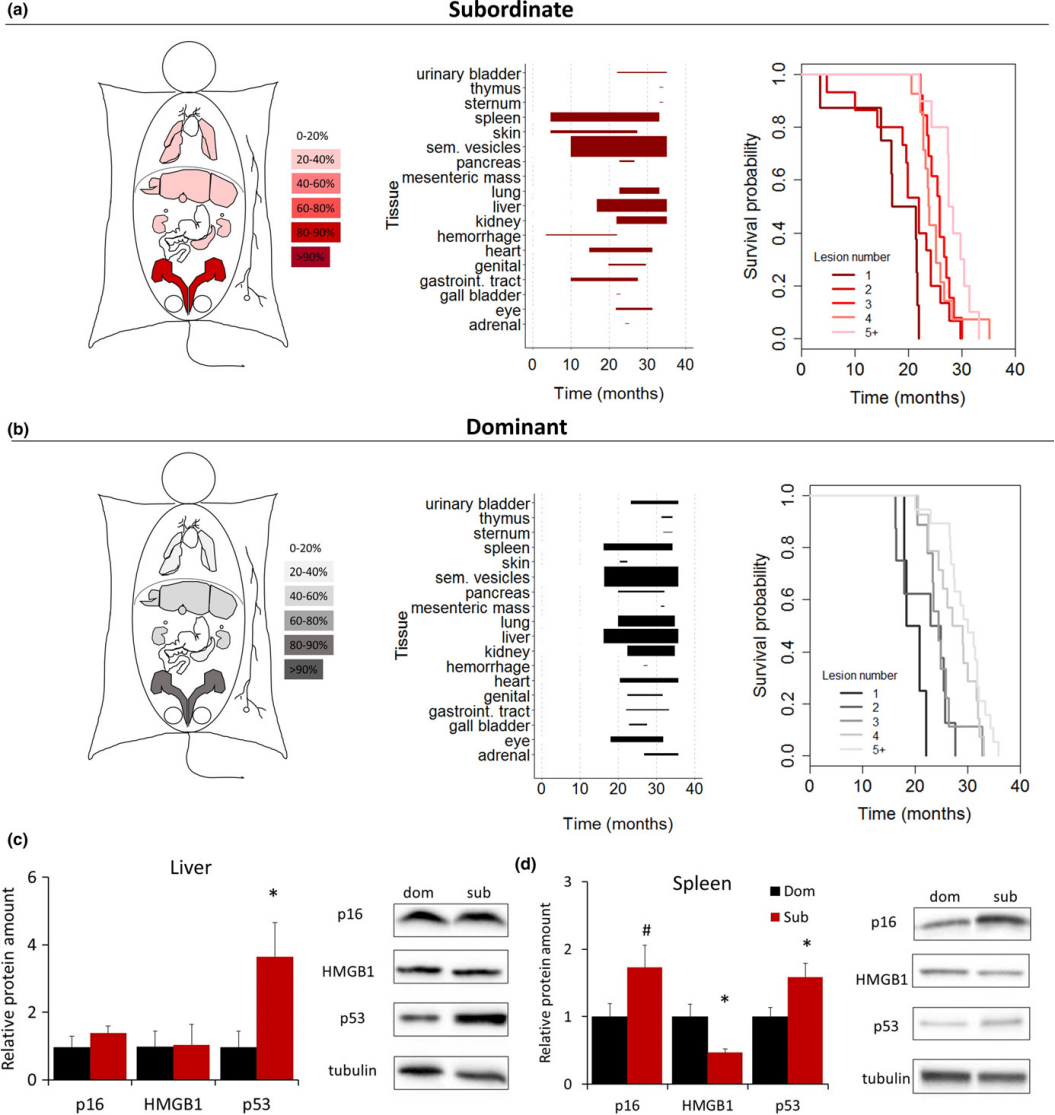
Achieved status affects the onset of aging‐associated organ lesions and cellular markers of senescence. (a, b) Heat map of organ‐specific lesions, age‐dependent distribution of macroscopic dissectible lesions in proportion to their prevalence within each organ, and survival probability as a function of the number of macroscopic dissectible lesions as detected at time of necropsy in dominant and subordinate mice. (c, d) Cell senescence markers in spleen and liver of dominant and subordinate mice [p16^Ink4a^, HMGB1 (high mobility group box 1), p53] *N* = 8/group. *p < .05, ^#^ *p* < .06. Data represent group mean** **±** **SEM

In an attempt to determine the cause of death, we conducted macroscopic evaluation of lesions detectable at necropsy on all mice. Macroscopic examination revealed that subordinate mice presented an earlier onset of dissectible lesions in most of the organs examined, a finding which is in line with their lower survival probability (Figure 4a,b). However, no organ‐specific prevalence could be identified to account for the differential mortality between the two groups (Figures 4a,b and S2). Interestingly, for both dominant and subordinate mice, the higher the number of lesions, the longer the lifespan, indicating an age‐related accumulation of nonlethal organ lesions and tumors (Figures 4a,b and S3).

To gain a mechanistic comprehension of this outcome, we conducted a second LCPS experiment in which subordinate and dominant mice were sacrificed when 17 months old (Figure S1e), an age chosen to anticipate the expected rapid decline in the survival curve typical of this strain (Baker et al., 2016; Mitchell et al., 2014; Turturro et al., 1999), allowing collection of well‐preserved organs for molecular and histopathological analyses. Firstly, we determined the expression of some of the classic cellular markers of senescence including p53, p16^Ink4a^, HMGB1, and telomere length (Baker et al., 2016; Campisi, 2013; Childs et al., 2017) in liver and spleen (organs chosen for showing the highest level of lesions, Figure S2). Subordinate mice showed a significant increase in p53, a tissue‐specific significant decrease in HMGB1, and a nearly statistically significant increase in p16^Ink4a^ when compared to dominant mice (Figure 4c,d), while telomere length was similar to the one from dominant subjects (Figure S4). Overall, these results are consistent with accelerated cellular senescence in subordinate mice.

### 2.5 Earlier onset but no increased frequency of tumors in subordinate mice

Given the known association between cellular senescence, cancer, and aging (Campisi, 2013), we asked whether the difference in social status during the LCPS led to differences in cancer prevalence that might underlie the survival effect. Although subordinate mice presented an earlier onset of tumors macroscopically detectable at autopsy, their incidence or spectrum was not different between the two groups, thus excluding solid tumors as a factor explaining the detrimental effect of achieved subordinate status on survival (Figure 4a,b; Figures S2 and S3a–c). Similarly, the analysis of sternum specimens, known to harbor most of the pathologies recognized as cause of death in C57BL/6J male mice (Brayton, Treuting & Ward, 2012), did not return any leukemia‐associated sign (Figure S3d–f), which was therefore discarded as a prominent cause of status‐dependent difference in survival.

### 2.6 Spontaneous occurrence of early‐stage atherosclerotic lesions in subordinate mice

**Figure 5:**
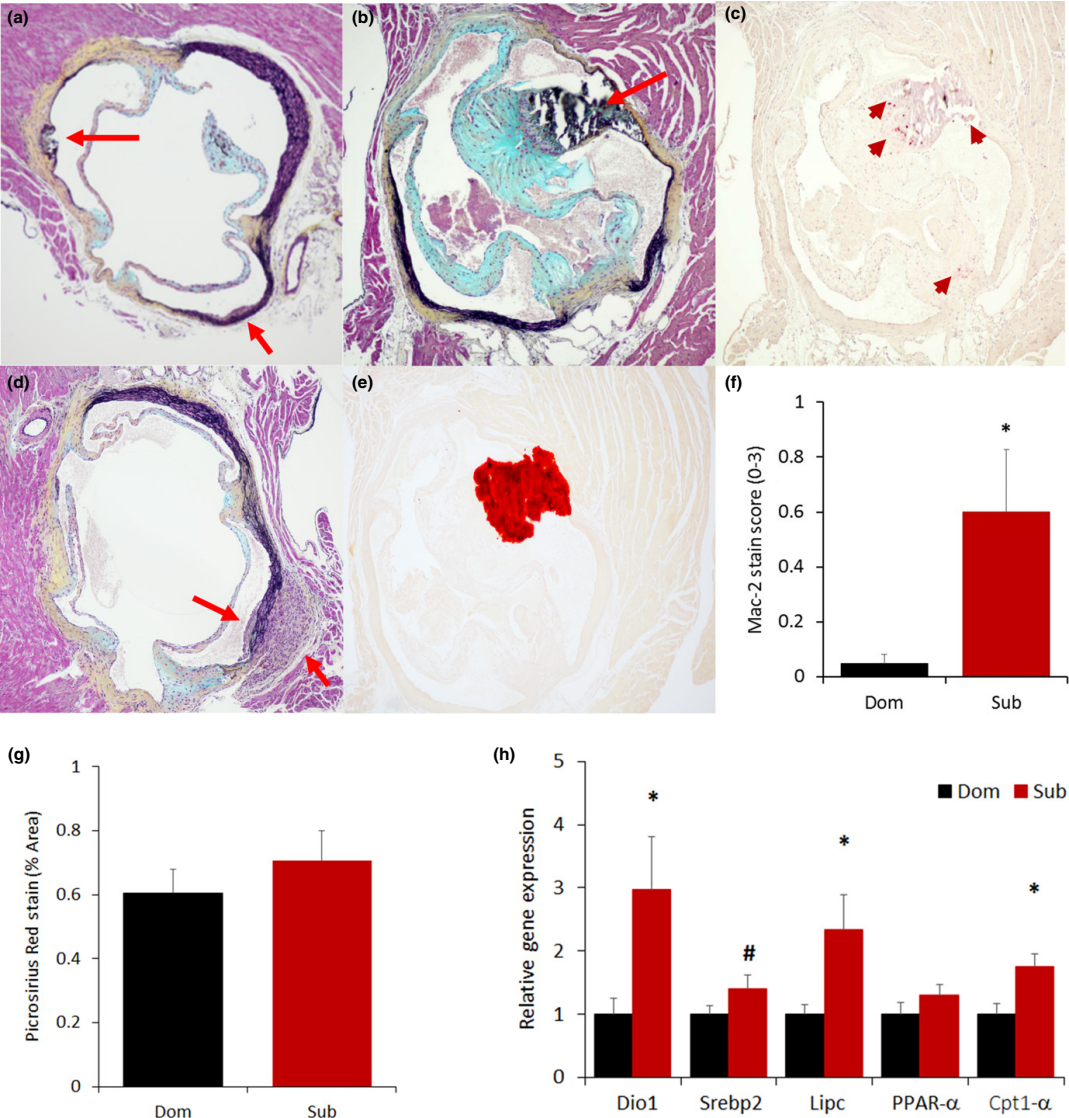
Early‐stage atherosclerotic lesions of the aortic sinuses are found exclusively in subordinate mice. Exemplar slides stained with Movat's staining showing the presence of rupture (a, top red arrow), immune cells infiltration (a, bottom red arrow, b and d) as well as calcification as confirmed by Alizarin stain (b, e). Inflammation was verified by Mac‐2 staining (c, red arrow) and quantified with a Mac‐2 score [f, *F*(1,14) = 5.76, *p* < .05]. (g) Quantification of fibrosis in the mouse myocardium using Picrosirius Red stain (representative images are shown in Figure S6). (h) Liver expression of lipid cholesterol metabolism markers [iodothyronine deiodinase 1 (Dio1), sterol regulatory element‐binding protein 2 (Srebp2), lipase C (Lipc), peroxisome proliferator‐activated receptor alpha (PPAR‐α), carnitine palmitoyltransferase 1α [(Cpt1α)]. *N* = 8/group *p < .05, ^#^ *p* < .07

Having ruled out a critical role for common tumors as the more likely explanation for the social status difference in survival, we focused on cardiovascular diseases based on their established association with stress, aging, and senescence (Costoli et al., 2004; Roth et al., 2015). We analyzed heart samples of LCPS mice sacrificed at 17 months of age to determine occurrence of myocardial damage or atherosclerosis. Remarkably, and unexpectedly, 50% of subordinate but none of the dominant mice manifested early‐stage commissural atherosclerotic lesions (Figures 5 and S5), while fibrosis was not different between the two groups (Figure 5g). Exclusive to subordinate, atherosclerotic lesions were mostly found in the region of the tunica media of the aortic sinus and presented a mature appearance as they were characterized by significantly increased immune cell infiltration (Figure 5c,f) as well as rupture, and calcification (Figure 5a,b,d,e). Consistently, subordinate mice showed increased expression of hepatic genes implicated in lipid and cholesterol metabolism when compared to dominant mice (Figure 5h).

Occurrence of atherosclerotic lesions in subordinate mice represents an unexpected and exceptional outcome (von Scheidt et al., 2017) suggesting that increased risk of cardiovascular disease is a critical pathogenic mechanism in relation to subordination stress‐induced decrease in lifespan.

## 3 DISCUSSION

Increased life stress and low SES are known risk factors for disease and reduced lifespan in humans (Marmot et al., 1991; Stringhini et al., 2017). Our study demonstrates a conserved role for social stress on shortening lifespan and increasing the risk of cardiovascular disease in a mouse model and identified a potential mechanistic link for this complex phenomenon.

Specifically, our model of LCPS identifies for the first time a causal relationship between the severity of the stressful environment (as quantified by aggressive behavior) and the individual coping style with reduced lifespan, suggesting a functional explanation to the concept of health disparity typical of low SES groups (Epel & Lithgow, 2014; Evans & Kim, 2010). Our results find support in results showing how the impact of stress on cardiovascular and psychiatric diseases is modulated by a passive or reactive coping style in response to stressful situations (Koolhaas et al., 1999). Consistently, subordinate male mice, corresponding to subjects characterized by high aggression received and low aggression exhibited, manifested HPA axis upregulation that is one of the common markers of chronic stress and allostatic load (McEwen, 1998; Seeman et al., 1997), which might set the stage for metabolic disease thus compromising healthspan. Subordinate mice also showed transient hyperglycemia and lifelong hyperphagia. The impact of hyperphagia on shortening lifespan of subordinate mice can be understood in light of the established effect on longevity attributed to calorie restriction (CR) (Balasubramanian, Howell & Anderson, 2017; Mitchell et al., 2014). Although the maximal benefits of CR depend on several contextual factors (i.e., species and strain (Ingram & de Cabo, 2017)), CR attenuates age‐associated metabolic diseases allowing to preserve metabolic flexibility and limiting lipodystrophy and hepatic metabolic consequences (Mitchell et al., 2014). It can be speculated then that the same mechanisms altered by CR to extend longevity might become altered in an opposing way in subordinate mice resulting in aberrant lipid metabolism and dyslipidemia (Sanghez et al., 2013, 2016), compromising metabolic efficiency and accelerating physical wasting, overall contributing to earlier mortality.

Subordination stress was also associated with the emergence of cellular senescence biomarkers such as increased p53 and decreased HMGB1 (and a nearly statistically significant increase in p16), but not others such as absolute telomere length. In landmark studies, Epel and colleagues (Blackburn, Epel & Lin, 2015; Epel et al., 2004) identified the association between exposure to life stress, shortening of leukocyte telomeres, and mortality in humans. The discrepancy with our current results could be attributed to intrinsic factors such as tissue specificity, species, and gender in the measure of telomere length (Sanders & Newman, 2013). Nevertheless, the combination of decreased HMGB1 (Davalos et al., 2013) and increased cell cycle inhibitors [p53 and p16^Ink4a^ (Childs et al., 2017)] are in line with the emergence of cellular senescence that can be associated with the development of tissue pathology and earlier mortality in subordinate mice. This hypothesis is supported by studies demonstrating that accumulation of senescent cells is causal in driving aging‐associated pathologies. For example, the ablation of senescent cells using the INK‐ATTAC or the p16‐3MR technologies, pharmacologically targeting the Ink4a transcriptionally active promoter in senescent cells, extended lifespan and improved healthspan in mice (Baker et al., 2011, 2016; Childs et al., 2016).

One of the major findings of our study is the occurrence of early‐stage atherosclerotic lesions in subordinate mice. Atherosclerosis represents an unexpected outcome in a species where this cardiovascular disease only develops in genetically predisposed strains under severe atherogenic dietary regimens (von Scheidt et al., 2017). Current and previous data point to hyperglycemia, insulin resistance, lipotoxicity, chronic low grade inflammation, dyslipidemia, and increased catecholamines and glucocorticoids as likely contributors to the development of early‐stage atherosclerosis in subordinate mice (Bartolomucci, 2007; Du et al., 2016; Heidt et al., 2014; Najafi et al., 2013; Razzoli, Karsten, Yoder, Bartolomucci & Engeland, 2014; Sanghez et al., 2013, 2016). Atherosclerosis and cardiovascular disease are among the major aging‐associated conditions in humans, and psychosocial factors such as job strain, low SES, and depression have been associated with increased risk of cardiovascular diseases and decreased lifespan (Kaplan & Manuck, 1999; Marmot et al., 1991; Rosengren et al., 2004; Shively, Register & Clarkson, 2009). Chronic stress can precipitate plaque progression in APOE−/− mice, increased sympathetic nervous system activation, HPA axis‐stimulated angiogenesis prompting inflammation with macrophage infiltration, and intraplaque hemorrhage (Heidt et al., 2014; Najafi et al., 2013; Wang et al., 2015). Notably, clearing senescent cells using either genetic approaches or senolytic drug treatments limits the development of atherosclerosis in the *Ldlr−/−* (Childs et al., 2017) or the APOE−/− (Wang et al., 2015) mouse models, a mechanism which is consistent with the phenotype of the subordinate mice and suggests a possible senescence‐associated link between stress and atherosclerosis.

While the full mechanistic comprehension of this phenomenon is beyond the scope of the present study, the evidence we presented composes a coherent picture of a metabolic dysregulation that follows the emergence of the acquisition of a low rank, subordinate status. A passive behavioral coping style in the face of lifelong psychosocial stress sets the stage for sustained hyperphagia and HPA hyperactivation overall leading to metabolic dysfunctions. Over time, these physiologic insults would favor the emergence of cellular senescence and predisposition to cardiovascular dysfunctions leading to reduced lifespan.

In conclusion, we developed a mouse model of lifelong chronic psychosocial stress and identified a causal link between high aggression received in the presence of a passive coping style as well as achieved subordinate status, and shorter lifespan in male mice. These findings provide a possible functional explanation to the established association between high stress exposure and low SES and lower survival in humans. Our data also suggest a mechanistic link between stress‐induced hyperphagia, chronically upregulated HPA axis as well as cellular senescence with cardiovascular disease, and reduced life expectancy in mice and, possibly, in humans.

## 4 MATERIALS AND METHODS

### 4.1 Animals

C57BL/6J (Jackson Labs), CD1 (Charles River Labs), and Sv129Ev (Taconic Farms) male mice were purchased at 10 weeks of age and housed at 12:12 hr light:dark cycle at 22 ± 2°C. At 12 weeks of age, mice were randomly allocated to the experimental conditions. The diets (Research Diets, Inc.) used in this study were as follows: a standard diet (D12405B), a high‐fat diet (D12451), and a mature rodent maintenance diet (D10012M). Cages contained corn cob bedding that was changed every 7 days. Cotton nestlets were placed in the cages for enrichment. Animal experiments were approved by the IACUC, University of Minnesota.

### 4.2 Lifelong chronic psychosocial stress model

The LCPS model consisted of a *baseline phase* of 5 days, during which all mice were singly housed all in one room, followed by a 4‐week *CPS phase* in which mice were exposed to daily defeats and sensory contact housing (enabled by a wire mesh partition bisecting the cage into two symmetrical compartments, each with food and water available at libitum), and an *aging phase* lasting until spontaneous death or moribund status (Figure 1a). In the aging phase, mice were housed in sensory contact thus experiencing a continued degree of threat stress related to the previous encounters.

The CPS was conducted essentially as previously described (Sanghez et al., 2013; Bartolomucci et al., 2009). Briefly each C57BL/6J (*N* = 172) male mouse, representing the experimental subject, was randomized using a simple randomization procedure of flipping a coin (Suresh, 2011) to be transferred as intruder to the home cage of either a CD1 (*N* = 86) or Sv129Ev (*N* = 86) resident mouse. The CD1 strain manifests high territorial aggression, while the Sv129Ev manifests a much lower level of territorial aggression and can thus be considered a weaker dominant (Bartolomucci et al., 2010; Dadomo et al., 2011; Zou et al., 2013). Resident and intruder mice were allowed to freely interact for a maximum of 10 min. After the interaction, resident and intruder mice were separated by a perforated partition, which allowed continuous sensory contact but no physical interaction. The partition was removed daily (between 8:30 and 9:30 a.m.), for a maximum of 10 min. During the social interaction, offensive behaviors of the animals were manually recorded. Furthermore, social status was determined as previously established. Specifically, subordinate social status was defined by the display of upright posture, flight behavior, and squeaking vocalization (Bartolomucci et al., 2010; Dadomo et al., 2011). After 1 week, half the dyads were randomized to a STD or a HFD using a simple randomization procedure of flipping a coin. Only dyads (*N* = 56 dominant and *N* = 61 subordinate) that reliably showed a stable dominant/subordinate hierarchy and in which the subordinate showed no attack after the fourth day of interaction were included in the analysis of the rank effect on healthspan and lifespan. As expected, most of the C57BL/6J acquiring a dominant status were paired with a Sv129Ev (43/56), while most of the C57BL/6J acquiring a subordinate status were paired with a CD1 (53/61).

After the conclusion of the CPS phase, the mice entered the aging phase of the study when each subordinate/dominant pair was kept in sensory contact in the same housing cage with the partition in place until spontaneous death occurred. The only procedures performed in this phase were the periodic healthspan assessments as detailed below. During the aging phase, all mice were fed standard diet. Finally, from the age of 10 months, all mice were switched to the mature rodent maintenance diet (D10012M) because of its better balance of essential nutrients tailored for aged rodents (Reeves, Nielsen & Fahey, 1993).

This study was designed to analyze the survival of the C57BL/6J mice as a function of a priori experimental variables such as pairing with either CD1 or Sv129Ev male mouse and diet, as well as a function of unbiased clustering of mice based on aggression received/exhibited. Furthermore, we used a design intended to expand upon past research which had examined the association with spontaneously achieved social status (dominant vs. subordinate) on healthspan (e.g., Bartolomucci et al., 2010; Sanghez et al., 2013) to examine the contribution of social status to longevity. The design employed a randomized experimental manipulation (described above) to increase the variability in achieved status thereby increasing the plausible variance in outcomes explained by the achieved status and, consequently, increasing statistical power and reducing the proportion of any relation between achieved status and subsequent outcomes due to potential confounding variables. In this study, we focus primarily on the proximal relation (and by implication plausible effect) of achieved status on healthspan and lifespan.

Mortality (age at death) was the study primary outcome. Animals were checked daily to determine vital status, and date of death was recorded to the nearest day. Mice found dead at each daily inspection were considered as censored deaths and were necropsied for tumor inspection. Criteria for euthanasia were based on an independent assessment made by an institutional veterinarian, and only cases where the condition of the animal was considered incompatible with continued survival are represented as deaths in the curves and censored to day of euthanasia. Conditions considered cause for euthanasia, as approved by the IACUC, University of Minnesota included the following: prolonged respiratory distress, tumor growth that impedes activity such as motion, eating or drinking, severe deformities or self‐mutilation, inability to eat or drink, or ataxia that prevents normal functions of eating and drinking.

### 4.3 Healthspan analysis

Body weight and food intake were measured weekly, while body composition (Echo MRI 3‐in‐1, Echo Medical System) and 4‐hr fasting glucose collected via tail snip (Accuceck‐Aviva, Roche) were recorded at baseline and at the end of the CPS phase. During the aging phase, the same health span parameters were assessed every 2 months, from 2 months up until 14 months after the end of the stress phase, and from then to completion every 4 months.

### 4.4 Corticosterone metabolite analysis

To accurately follow the secretion of glucocorticoids in our mice without interfering with the activation of the HPA axis by repeated handling and blood sampling, a noninvasive technique was applied to monitor adrenocortical activity by measuring corticosterone metabolites (CM) in the feces of mice. This technique of glucocorticoid metabolite quantification in fecal samples has been established in an increasing number of species and has been extensively validated for laboratory mice (Touma, Palme & Sachser, 2004). Fecal samples were collected 2 months after the end of the CPS and were analyzed for immunoreactive corticosterone metabolites using a 5α‐pregnane‐3β,11β,21‐triol‐20‐one enzyme immunoassay (EIA) according to published methods (Heinzmann et al., 2014). Before EIA analysis, the fecal samples were dried (2 hr at 80°C), homogenized, and aliquots of 0.05 g of powdered feces were extracted with 1 ml of 80% methanol. The EIA was performed on protein A‐coated microtitre plates. After overnight incubation (at 4°C) of standards (range: 0.8–200 pg/well) and samples with steroid antibody and biotinylated label, the plates were emptied, washed, and blotted dry, before a streptavidin–horseradish peroxidase conjugate was added. After 45‐min incubation time, plates were emptied, washed, and blotted dry. The substrate (tetramethylbenzidine) was added and incubated for another 45 min at 4°C before the enzymatic reaction was stopped with 1 mol/L sulfuric acid. Then, the optical density (at 450 nm) was recorded with an automatic plate reader, and the steroid concentrations were calculated. The intra‐ and interassay coefficients of variation were 8.8% and 13.4%, respectively.

### 4.5 Senescence biomarkers and pathological analysis study

In a second experiment, 70 C57BL/6J, 35 CD1, and 35 Sv129Ev mice purchased from the respective vendor at 10 weeks of age and exposed to LCPS. Of these 70 C57BL/6J, 21 achieved dominant (all mice paired with a Sv129Ev) and 31 achieved subordinate social status (26/31 when paired with a CD1). Mice were sacrificed at 17 months of age for the collection of tissue specimens, at which time they were in apparent good health as this age precedes the rapid decline of the lifespan curve typical of this strain. These animals are not included in the survival analysis of the lifespan study. Mice were euthanized by CO_2_ inhalation in the morning (between 8 and 10 a.m.). The dissection was performed as rapidly as possible following euthanasia. Major organs were removed, cut into appropriate size pieces, and either flash‐frozen in liquid nitrogen, or placed in 4% PFA for preservation. Flash‐frozen tissue samples were stored at −80°C. After several days of PFA fixation at room temperature, tissue fragments were transferred to 70% ethanol and stored at 4°C. Specimens of sternum were decalcified for 3 hr by “Decalcifying Solution‐Lite” (Sigma‐Aldrich, St. Louis, MO, USA). All dissectible tumors were analyzed and eight animals per group were randomly selected to conduct the senescence markers, heart morphology, and immunohistochemistry analyses as detailed below.

### 4.6 Histological analysis

Fixed lung, liver, and sternum samples were embedded in paraffin, and 4‐μm‐thick sections were dewaxed in xylene and rehydrated through graded alcohol to water and stained with hematoxylin and eosin. Histopathological analysis of tumors was conducted by a certified pathologist blinded to animal's condition of social status.

The heart morphometric analysis and IHC were conducted at the University of Washington Pathology Core facility. Formalin‐fixed hearts were cut horizontally at midventricular level into two halves and paraffin embedded. The upper part was consecutively cut and all sections collected. Next, six 4‐μm heart sections covering 100 μm of aortic sinuses were selected and stained with Movat, Alizarin Red S, and Mac‐2 staining protocols. To identify calcium in tissue sections, a standard protocol for Alizarin Red S staining was used. Calcium forms an Alizarin Red S‐calcium complex in a chelation process, and the orange‐red product is birefringent. The rat anti–Mac‐2 antibody was used (1:3,000, Cedarlane; Hornby, Ontario, Canada) to detect infiltrating monocytes/macrophages. For each animal, the aortic sinuses were photographed under 100× magnification and quantified by computer image analysis (imagepro plus image analysis software) for lesion size and calcium content. A degree of macrophage infiltration was semiquantitatively scored from 0 to 3. Additionally, a 4‐μm midventricular cross sections of myocardium stained with Picrosirius Red stain (PSR) (Direct Red 80, Sigma, Saint Louis, MO, USA) were analyzed for fibrosis. Stained sections were photographed using standard and polarized light for birefringence properties and morphometrically measured.

### 4.7 Absolute telomere length

Absolute telomere length was determined according to published protocol (O'Callaghan & Fenech, 2011). DNA from spleen and liver was isolated using DNeasy Blood and Tissue kit (Qiagen) with slight modification of adding 1 mm DTT to eluate. Briefly, a standard curve of known quantities of a synthesized 84 mer oligonucleotide containing only TTAGGG repeats was generated based on CT (cycle threshold) using iQ SYBR Green Supermix (Bio‐Rad). A standard curve using the single copy gene, 36B4, was generated to serve as a control for amplification and to determine genome copies per sample. For both standard curves using synthesized oligomers, 20 ng of plasmid DNA (pBR322 Vector, New England Biolabs) was loaded in all wells. For samples, 20 ng of DNA was loaded in triplicate. Synthesized oligomers and primer sequences for qPCR in Table S2 and all samples were processed on a 96CFX real‐time system (Bio‐Rad).

### 4.8 Quantitative real‐time polymerase chain reaction

Quantitative real‐time polymerase chain reaction was conducted according to standard protocols (Razzoli et al., 2016). Table S2 lists primer information. The best keeper was generated from beta‐actin and TFIIB reference genes (Pfaffl, Tichopad, Prgomet & Neuvians, 2004) and used to normalize target gene values according to the comparative threshold ΔCT. Gene expression data are presented as normalized linear‐transformed values (2‐ΔCT) relative to the dominant group.

### 4.9 Western Blot

Protein lysates were obtained by homogenizing tissue in RIPA buffer (50 mm Tris [pH 7.5], 1 mm EDTA, 1% Triton X‐100, 0.1% sodium deoxycholate, 0.1% SDS, 50 mm NaCl). 25 μg of protein was resolved on 4%–15% acrylamide gels, transferred to PVDF membranes, and probed overnight with primary antibodies: HMBP1 (3935S), p53 (32532S), and tubulin (2146S) from Cell Signaling and p16 (Abcam, Ab51243). Membranes were incubated with goat anti‐rabbit‐HRP secondary antibody (Santa Cruz Biotechnology, sc‐2004) and then ECL was applied to generate signal and imaged with ChemiDox MP imaging system (Bio‐Rad). Intensities of protein detection were quantified using imagej2.

### 4.10 Statistical analysis

The survival analyses were implemented in R Studio from scratch using the methodological references given below. Survival probability was assessed through the use of the nonparametric log‐rank (Mantel‐Haenszel) test to compare the differences in Kaplan–Meier survival curves using the “survival” package of the R language. When significant, binary comparisons were conducted using Bonferroni corrected log‐rank test performed between two groups of interest at a time. The same survival package was used to fit the Cox proportional hazard model to actual lifespans to calculate the hazard ratio (HR). Median and maximum survival were assessed using Fisher's exact test to determine statistical significance. Maximum lifespans were calculated as the proportion of either dominant or subordinate mice still alive when the total population reached 90% mortality (Hofmann et al., 2015). Cluster analysis was conducted on the data of aggression exhibited and received by the experimental subjects that were partitioned through k‐means clustering; Ward's method was then used for hierarchical clustering based on a Euclidean distance matrix using the “cluster” package of the R language. Food intake, body weight, body composition (fat mass and fat‐free mass), and glucose were analyzed with a two‐way analysis of variance (ANOVA) design (main factors: achieved social status and diet) and with a two‐way ANOVA design for repeated measures (main factors: achieved status and diet by time) with Tukey's post hoc comparisons for binary comparisons. Bonferroni correction was applied for repeated comparisons between groups. Comparisons was applied for the histopathology and molecular data were performed using unpaired *t* tests. A *p* value of ≤.05 was considered statistically significant for these outcomes. Analyses were performed using R (Version 0.99.891—© 2009‐2016 RStudio, Inc.) or Statistica 13.0 (Dell Inc., Tulsa, OK). Data are expressed as means ± standard error of the mean (*SEM*).

## CONFLICT OF INTEREST

None declared.

## AUTHOR CONTRIBUTIONS

M.R., K.D., C.E., J.M., and N.S. conducted the in vivo experiments. A.G., M.M., and J.P. performed in vitro experiments. M.K. and E.A.P. conducted the histological evaluation of the tumors in consultation with D.A.L.; C.T. and R.P. conducted the fecal corticosterone metabolites analysis; M.R. analyzed the data with the help of A.B.; A.B. conceived the project with the help of M.R. and D.B.A.; A.B. and M.R. wrote the manuscript.
