# Correction to “Social Stress Shortens Lifespan in Mice”

**DOI:** 10.1111/acel.70604

**Published:** 2026-06-25

Razzoli, M. , K. Nyuyki‐Dufe , A. Gurney , et al. 2018. “Social Stress Shortens Lifespan in Mice.” Aging Cell 17: e12778. 10.1111/acel.12778.29806171 PMC6052478

The antibody Abcam's ab51243 that is specific for p16‐ARC and not for p16^Ink4a^ was erroneously used. This was an unwanted incorrect selection of the antibody since the intent was to measure the p16^Ink4a^ protein.

The results using this antibody did not highlight a significant difference in the liver, and only a non‐significant trend for an increase was observed in the spleen of subordinate mice. Therefore, no influential conclusion was drawn based on the use of an inappropriate antibody selection. The conclusion that subordination stress increased markers of cellular senescence was based on a significant change in other markers such as p53 and HMGB1.

Accordingly, the Figure 4 legend, subsection 2.4, the Discussion, and subsection 4.9 should be corrected as follows:

In Figure 4 legend, the text “(c, d) Cell senescence markers in spleen and liver of dominant and subordinate mice [p16^Ink4a^, HMGB1 (high mobility group box 1), p53] *N* = 8/group. *p < 0.05, ^#^ *p* < 0.06. Data represent group mean ± SEM.” was incorrect. Figure 4 and its correct legend are as follows:

**Figure:**
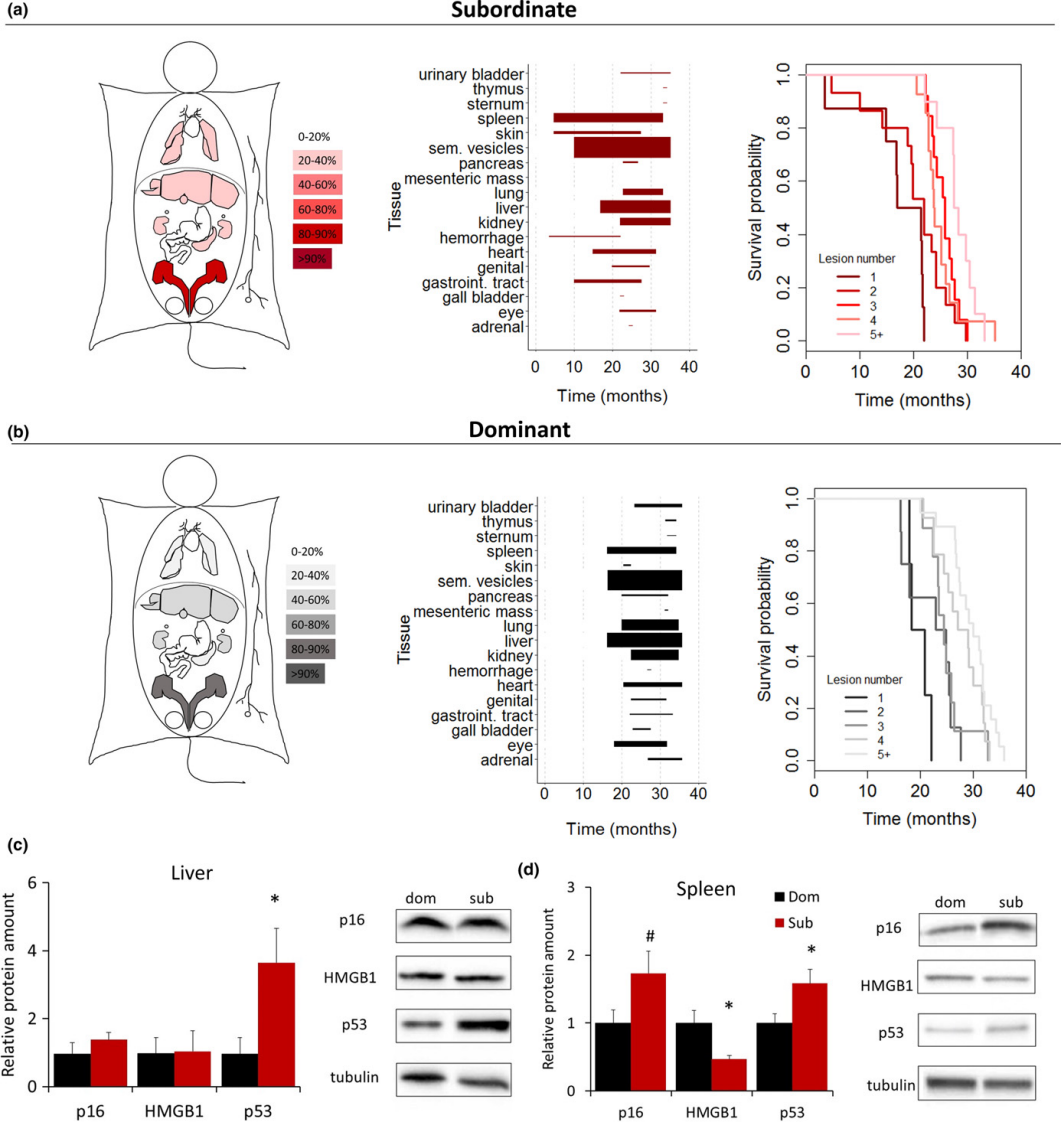
**FIGURE 4** Achieved status affects the onset of aging‐associated organ lesions and cellular markers of senescence. (a, b) Heat map of organ‐specific lesions, age‐dependent distribution of macroscopic dissectible lesions in proportion to their prevalence within each organ, and survival probability as a function of the number of macroscopic dissectible lesions as detected at time of necropsy in dominant and subordinate mice. (c, d) Cell senescence markers in spleen and liver of dominant and subordinate mice [HMGB1 (high mobility group box 1), p53] *N* = 8/group. *p < 0.05, ^#^ *p* < .06. Data represent group mean ± SEM

In subsection 2.4, the text “Firstly, we determined the expression of some of the classic cellular markers of senescence including p53, p16^Ink4a^, HMGB1, and telomere length (Baker et al. 2016; Campisi 2013; Childs et al. 2017) in liver and spleen (organs chosen for showing the highest level of lesions, Figure S2). Subordinate mice showed a significant increase in p53, a tissue‐specific significant decrease in HMGB1, and a nearly statistically significant increase in p16^Ink4a^ when compared to dominant mice (Figure 4c,d), while telomere length was similar to the one from dominant subjects (Figure S4).” was incorrect. It should read as follows:

“Firstly, we determined the expression of some of the classic cellular markers of senescence including p53, HMGB1, and telomere length (Baker et al. 2016; Campisi 2013; Childs et al. 2017) in liver and spleen (organs chosen for showing the highest level of lesions, Figure S2). Subordinate mice showed a significant increase in p53, a tissue‐specific significant decrease in HMGB1 when compared to dominant mice (Figure 4c,d), while telomere length was similar to the one from dominant subjects (Figure S4).”

In the Discussion, the text “Subordination stress was also associated with the emergence of cellular senescence biomarkers such as increased p53 and decreased HMGB1 (and a nearly statistically significant increase in p16), but not others such as absolute telomere length. […] Nevertheless, the combination of decreased HMGB1 (Davalos et al. 2013) and increased cell cycle inhibitors [p53 and p16^Ink4a^ (Childs et al. 2017)] are in line with the emergence of cellular senescence that can be associated with the development of tissue pathology and earlier mortality in subordinate mice.” was incorrect. It should read as follows:

“Subordination stress was also associated with the emergence of cellular senescence biomarkers such as increased p53 and decreased HMGB1, but not others such as absolute telomere length. […]. Nevertheless, the combination of decreased HMGB1 (Davalos et al. 2013) and increased cell cycle inhibitor p53 (Childs et al. 2017) are in line with the emergence of cellular senescence that can be associated with the development of tissue pathology and earlier mortality in subordinate mice.”

In subsection 4.9, the text “p16 (Abcam, Ab51243).” was incorrect. It should read: “p16‐ARC (Abcam, Ab51243).”

We apologize for these errors.

