# Supplementary material for: Social stress shortens lifespan in mice
Source: Aging Cell. 2018 May 28;17(4):e12778. doi: 10.1111/acel.12778 (PMC6052478; doi:10.1111/acel.12778)
Supplement: Supplementary file 1 [file ACEL-17-na-s001.docx]

**Supplementary figures**


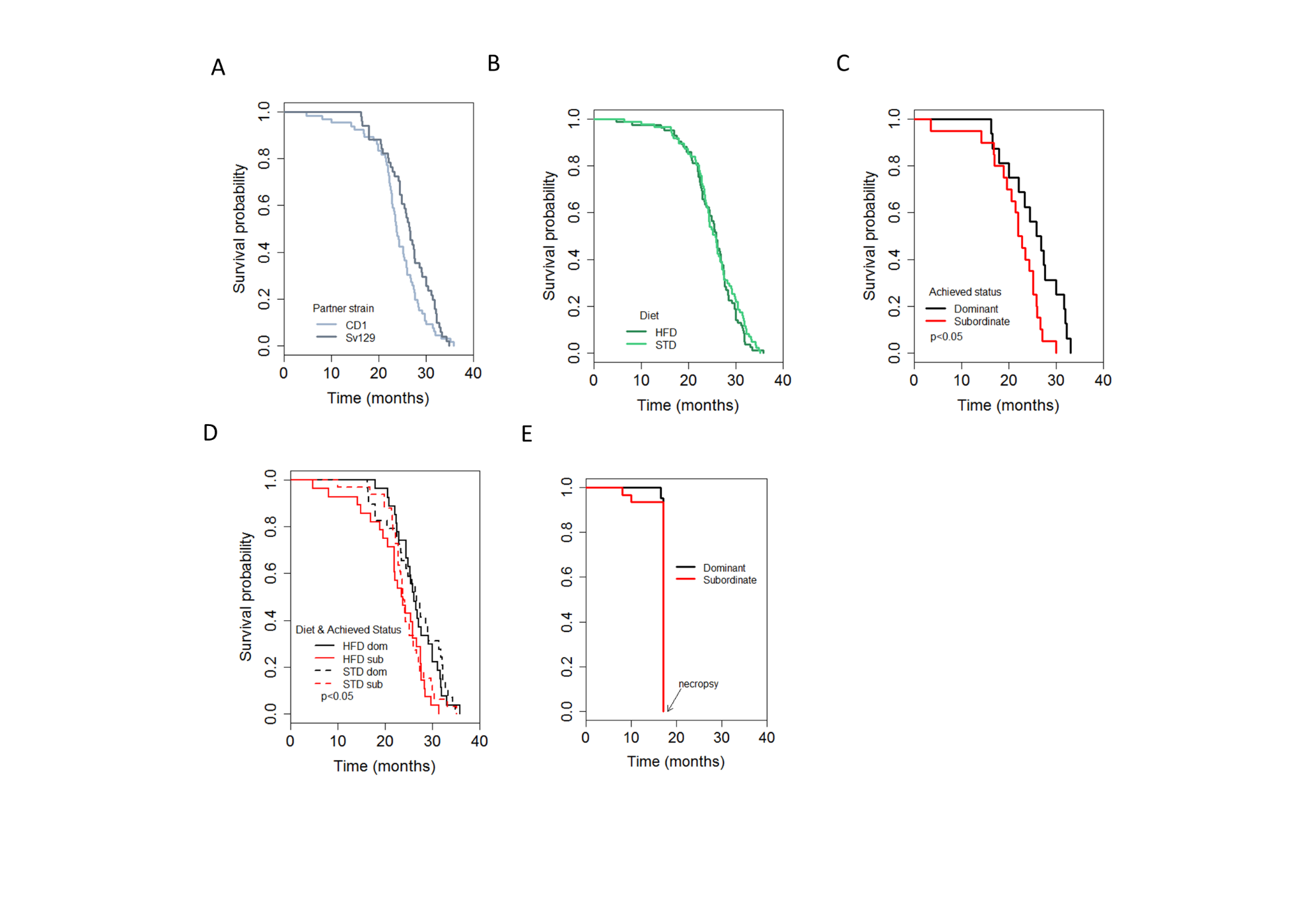


Fig. S1. Survival analysis. Survival curve in consideration of (A) the intent to treat (ITT) as represented by the strain of the resident mouse randomly assigned to each C57BL/6J experimental subject and (B) the diet (standard diet, STD; high fat diet, HFD). (C) Survival curve of the mice found spontaneously dead analyzed according to their achieved status (log-rank test, Chisq= 6.1 on 1 degrees of freedom, p< 0.05; Bonferroni corrected p value for binary comparisons = 0.0125). (D) Survival curve in consideration of the interaction between the diet and the achieved status (log-rank test, Chisq= 9.8 on 3 degrees of freedom, p< 0.05). E) Survival analysis of the senescence biomarkers and pathological analysis study in which subordinate and dominant mice were euthanized at approximately 17 months of age.


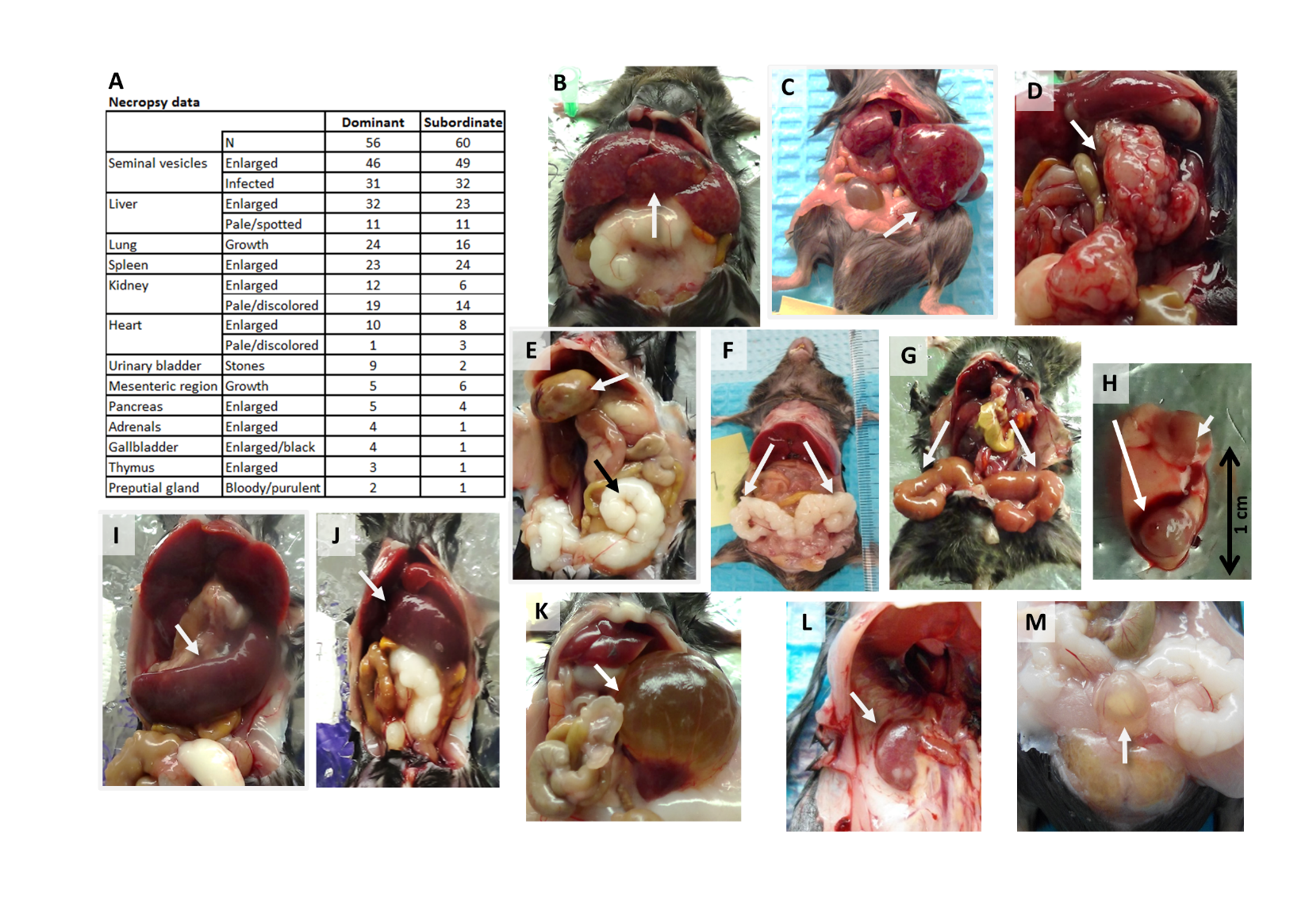
Fig. S2 Summary of macroscopic findings at necropsy. (A) Table of occurrence of lesions detected at necropsy in Dominant and Subordinate mice. Representative picture of lesions: (B) multifocal hepatic necrosis; (C) focal nodular hepatic lesion; (D) pancreas lesion; (E) enlarged gallbladder (white arrow) and enlarged seminal vesicles (black arrow); (F) enlarged seminal vesicles; (G) infected seminal vesicles; (H) pulmonary neoplastic foci; (I) splenomegaly; (J) splenomegaly; (K) renal pelvis enlargement and cystic dilatation; (L) multifocal suppurative embolic nephritis; (M) urinary bladder stones.

**
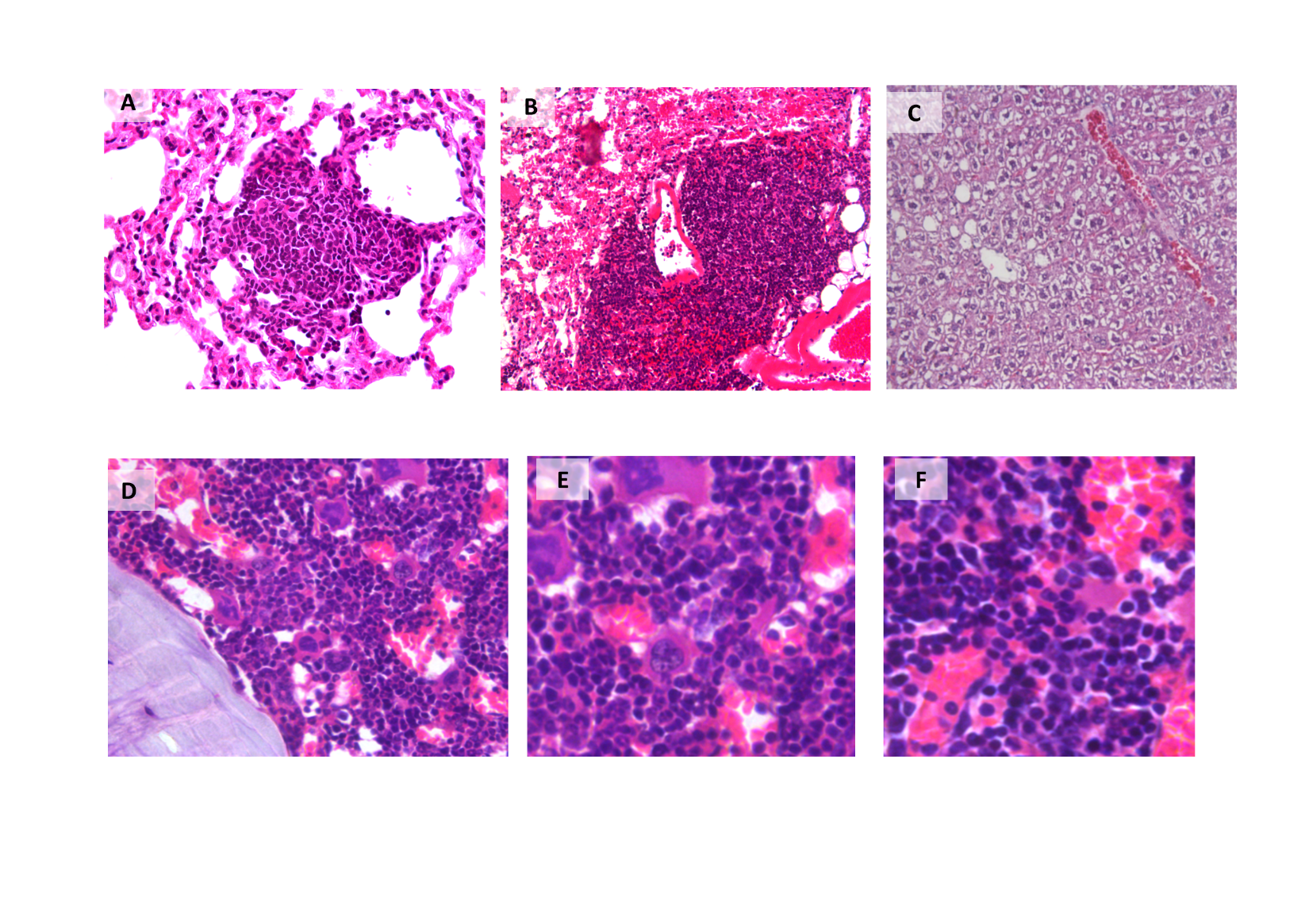
**

**Fig. S3. Histopathology of representative tumors collected from mice at 17 months of age and non-neoplastic bone marrow from sternum samples:** (**A**) lung with aggregation of hyperchromatic cells; (**B**) lung with focal and severe lymphocyte infiltration; (**C**) liver hepatocytes with atypia; sternum samples from representative subordinate (D,E) and dominant (F) mice (100x-400X).


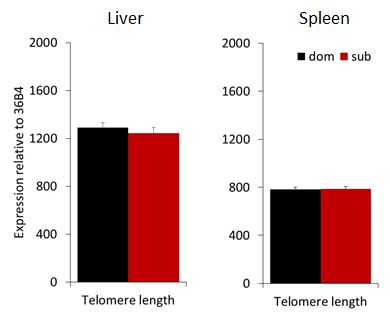


**Fig. S4 Absolute telomere length.** Absolute telomere length was measured in the liver and spleen of Dominant and Subordinate mice sacrificed at 17 months of age. N=8/group.


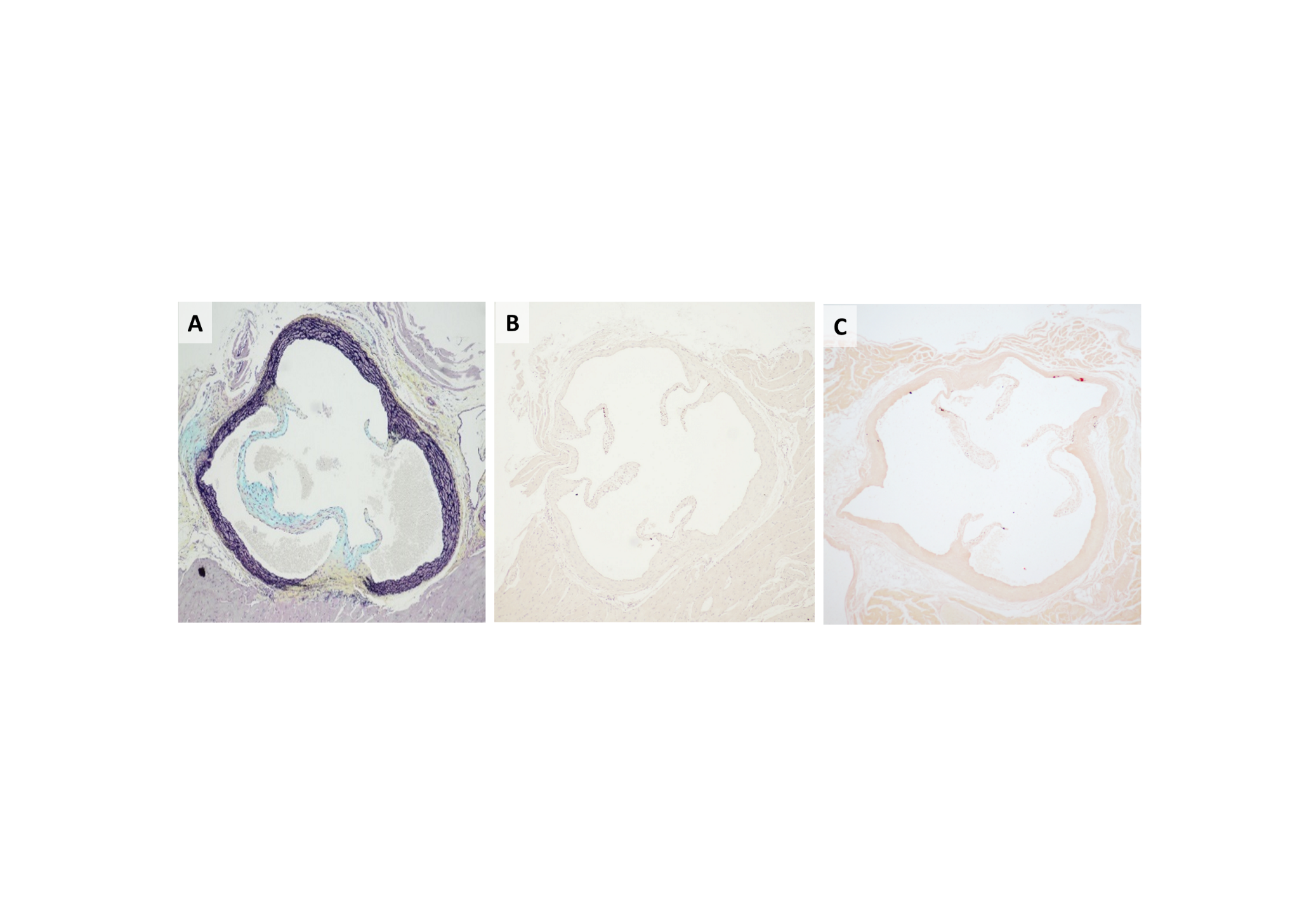


**Fig. S5 Dominant mice sacrificed at 17 months of age did not present signs of atherosclerotic lesions in aortic sinuses.** Exemplar sections from a dominant C57BL/6J subject (A) Movat’s staining, (B) Mac-2 staining, (C) Alzarin staining.


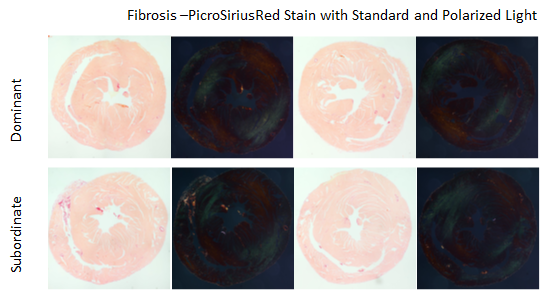


Fig. S6. Cardiac fibrosis in Dominant and Subordinate mice sacrificed at 17 months of age. A) Representative Picro-Sirius Red Stain (Connective Tissue Stain) used for visualization of collagen I and III fibers in addition to muscle in tissue sections. The PSR stain may be viewed using standard light microscopy or polarized light resulting in birefringence of the collagen fibers to distinguish between type I and type III. The quantification of the fibrosis is presented in Figure 5. N=8/group.

Table S1. Oligomers used to quantify telomere length in mouse.

| Standards | Oligomer Name | Oligomer sequence (5′-3′) | Amplicon size (bp) |
| --- | --- | --- | --- |
|  | Telomere Standard | (TTAGGG)14 | 84 |
|  | 36B4 standard | CAGCAAGTGGGAAGGTGTAATCCGTCTCCACAGACAAGGCCAGGACTCGTTTGTACCCGTTGATGATAGAATGGG | 75 |
| qPCR primers | Oligomer Name | Oligomer sequence (5′-3′) | Amplicon size (bp) |
|  | teloF | CGGTTTGTTTGGGTTTGGGTTTGGGTTTGGG TTTGGGTT | >76 |
|  | teloR | GGCTTGCCTTACCCTTACCCTTACCC TTACCCTTACCCT |  |
|  | 36B4F | ACTGGTCTAGGACCCGAGAAG | 78 |
|  | 36B4R | TCAATGGTGCCTCTGGAGATT |  |

Table S2.

**Primer sequence of target and reference genes used for qPCR.**

| Gene | sequence (5′-3′) |
| --- | --- |
| Beta-actin | F: GGC ACC ACA CCT TCT ACA ATG  R: GGG GTG TTG AAG GTC TCA AAC |
| TFIIB | F: GAA TTG CCA AAC TCA TCA AAA CT  R: TGG AGA TTT GTC CAC CAT GA |
| Ppar-alpha | F: CAT TGT GTG ACA TCC CGA CAG  R: ATT CGG CTG AAG CTG GTG TAC |
| Dio1 | F: CCACCTTCTTCAGCATCC R: AGTCATCTACGAGTCTCTTG |
| Srebp2 | F: CCAAAGAAGGAGAGAGGCGG R: CGCCAGACTTGTGCATCTTG |
| Lipc | F: TTCTCGGAGCAAAGTTCACCTAA R: CCAGCCCTGTGATTCTTCCA |
| Ctp1-alpha | F: CCTGCATTCCTTCCCATTTG R: TTGCCCATGTCCTTGTAATGTG |
